# Zhizhu Kuanzhong, a traditional Chinese medicine, alleviates gastric hypersensitivity and motor dysfunction on a rat model of functional dyspepsia

**DOI:** 10.3389/fphar.2022.1026660

**Published:** 2022-11-17

**Authors:** Zhuanglong Xiao, Jing Xu, Jun Tan, Shengyan Zhang, Nian Wang, Ruiyun Wang, Pengcheng Yang, Tao Bai, Jun Song, Zhaohong Shi, Wenliang Lyu, Lei Zhang, Xiaohua Hou

**Affiliations:** ^1^ Department of Gastroenterology, Union Hospital, Tongji Medical College, Huazhong University of Science and Technology, Wuhan, China; ^2^ Clinical College of Chinese Medicine, Hubei University of Chinese Medicine, Wuhan, China; ^3^ Department of Chinese Medicine, Hubei College of Chinese Medicine, Jingzhou, China; ^4^ Department of Gastroenterology, The First Hospital of Wuhan (Wuhan Integrated TCM and Western Medicine Hospital), Wuhan, China; ^5^ Department of Gerontology, Union Hospital, Tongji Medical College, Huazhong University of Science and Technology, Wuhan, China

**Keywords:** Zhizhu Kuanzhong capsule, functional dyspepsia, gastrointestinal motility, greater splanchnic afferents, 5-hydroxytryptamine

## Abstract

**Ethnopharmacological relevance:** Zhizhu Kuanzhong (ZZKZ) is a traditional Chinese medicine modified from classic formula Zhizhu decoction in “Synopsis of Golden Chamber” (Han Dynasty in the 3^rd^ century) and the Zhizhu pill in “Differentiation on Endogenous” in Jin Dynasty (1,115–1,234). ZZKZ contains four botanical drugs, including *Citrus × Aurantium* L [Rutaceae; *Aurantii Fructus Immaturus*], *Atractylodes Macrocephala* Koidz. [Compositae; *Rhizoma Atractylodis Macrocephalae*], *Bupleurum Chinense* DC [Apiaceae; *Radix Bupleuri Chinensis*], and *Crataegus Pinnatifida* Bunge [Rosaceae; *Fructus Crataegi Pinnatifidae*], which have been widely used in clinical therapy for functional dyspepsia (FD).

**Aim of the study:** This study aimed to evaluate the pharmacological effects and mechanisms of action of ZZKZ on gastric hypersensitivity and motor dysfunction in a rat model of FD.

**Materials and methods:** FD was induced in Sprague-Dawley rats by neonatal gastric irritation with 0.1% iodoacetamide. The FD rats were treated with ZZKZ (0.5 g/kg, 1.0 g/kg, or 1.5 g/kg respectively) by gavage for 7 days, while domperidone (3 mg/kg) acted as treatment control. Body weight gain, food intake, gastric emptying, and intestinal propulsion were also measured. *Ex vivo* gastric smooth muscle activity recordings and greater splanchnic afferent (GSN) firing recordings were employed to evaluate gastric motility and sensation. Particularly, the role of 5-HT in the action of ZZKZ in improving gastric dysmotility and hypersensitivity was explored.

**Results:** ZZKZ promoted weight gain, food intake, gastric emptying, and intestinal propulsion in FD rats. ZZKZ promoted spontaneous and ACh-induced contractions of gastric smooth muscle strips in FD rats, alleviated spontaneous activity, and chemical (acid perfusion) and mechanical (intragastric distension) stimulated GSN firing in FD rats. ZZKZ ameliorated gastric smooth muscle contraction and GSN firing induced by 5-HT in FD rats. ZZKZ stimulated the release of serum 5-HT, with reduced 5-HT_3_ receptor and increased 5-HT_4_ receptor mRNA expression in the guts of FD rats.

**Conclusion:** This study demonstrated that ZZKZ improves FD-related gastric hypersensitivity and motor dysfunction and should be an effective compound for relieving FD symptoms. The gastric 5-HT system with lower 5-HT_3_ activity and increased 5-HT_4_ distribution is involved in the mechanisms of ZZKZ underlying the treatment of FD.

## Graphical Abstract

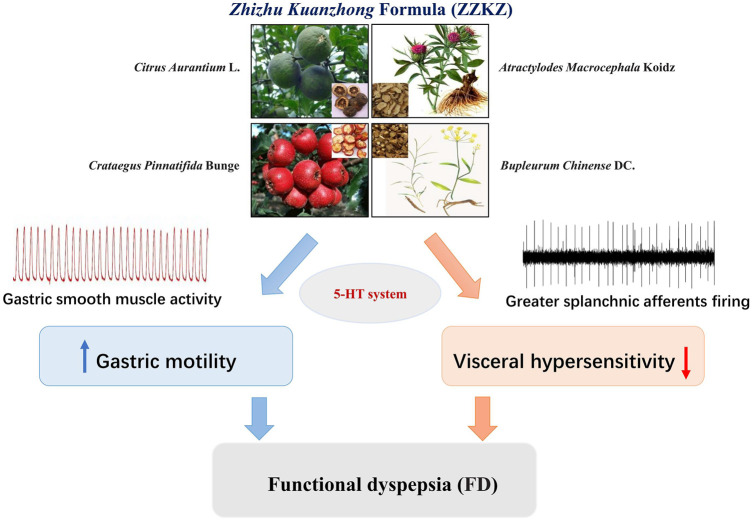

## 1 Introduction

Functional dyspepsia (FD) is a complex symptom referable to the gastroduodenal region of the gut and is characterised by epigastric pain or burning, postprandial fullness, and/or early satiety (Wauters et al., 2020). FD affects up to 16% of individuals in the general population (Ford et al., 2020) and comprises subtypes of postprandial distress syndrome (PDS), epigastric pain syndrome (EPS), and overlapping of the two subtypes according to the Rome IV criteria (Talley et al., 2016). FD symptoms can be caused by disturbed gastric motility (eg. delayed gastric emptying or inadequate fundic accommodation), gastric sensation (eg. hypersensitivity to gas and bloating), gastroduodenal inflammation, and psychiatric comorbidity (Sayuk and Gyawali, 2020; Wauters et al., 2020). Thus, pharmacological therapy is mostly based on the subtype of FD, including prokinetic and fundus-relaxing drugs for PDS and acid-suppressive drugs for EPS. However, multiple studies have shown that different pathophysiological mechanisms could contribute to all subtypes (Tack et al., 2004; Tack et al., 2006; Enck et al., 2017). Single-targeted treatments can be challenging in treatment of FD with multiple etiologies. Therefore, more and more multipotent herbal preparations are being proposed across countries.

Traditional Chinese medicine (TCM) theory and natural herbal medicines have been widely used in the initial treatment of functional gastrointestinal disorders for a long time (Sankararaman et al., 2022). Multi-component medicinal herbs usually have broad pharmacological applications targeting to multiple etiologies which hit multiple targets of FD and then exert a synergistic therapeutic action (Yang et al., 2014; Peng et al., 2022). The Zhizhu Kuanzhong (ZZKZ) capsule is a TCM originating from two classical formulas: the Zhizhu decoction in the “Synopsis of the Golden Chamber’’ (Han Dynasty in the 3^rd^ century) and the Zhizhu pill in the “Differentiation on Endogenous” in Jin Dynasty (1,115–1,234) (Zhang et al., 2012). ZZKZ contains four botanical drugs, including *Aurantii Fructus Immaturus*, *Rhizoma Atractylodis Macrocephalae*, *Radix Bupleuri Chinensis,* and *Fructus Crataegi Pinnatifidae* (Zhang et al., 2012). More and more effective components and functions of each ingredients of ZZKZ have been elucidated by phytochemical and pharmacological studies (Jurikova et al., 2012; Feng et al., 2020; Chang et al., 2022; Jia et al., 2022). As the preferred TCM in regulating gastrointestinal motility and psychiatric symptoms, ZZKZ has been widely used in patients with FD (Guo et al., 2011; Lin et al., 2018; Wen et al., 2019; Xiao et al., 2022; Xu et al., 2022), especially those with the PDS subtype, with the differentiation of TCM syndrome being Spleen-deficiency and Qi-stagnation (Lin et al., 2018). A multicentre randomised controlled trial demonstrated the effectiveness of ZZKZ capsules in treating PDS (Xiao et al., 2019). However, the underlying mechanism of action of ZZKZ remains unclear.

Gastric motor dysfunction and visceral hypersensitivity are the most widely accepted aetiopathogenesis of FD symptoms (Vanheel and Farre, 2013) Delayed gastric emptying, a common motility disturbance in patients with FD, is closely related to abnormal smooth muscle contraction, especially in the gastric antral smooth muscle (Vanheel and Farre, 2013; Xiong et al., 2015) In addition, hyperreactivity and plasticity of the greater splanchnic afferent nerves (GSN), which innervate the stomach and perceive pain and noxious stimuli, are correlated with FD symptoms, including gastric hypersensitivity (Holzer, 2004; Liu et al., 2008). Moreover, 5-hydroxytryptamine (5-HT) plays an important role in the regulation of gastrointestinal sensation and smooth muscle movement (Keating and Spencer, 2019). It is widely believed that 5-HT is primarily synthesized in intestinal mucosal enterochromaffin cells *via* tryptophan hydroxylase 1 (TPH1) (Li et al., 2011; Gershon, 2013). Decreased serum 5-HT levels have been found in patients with FD, which is associated with impaired gastric motility and visceral allergic symptoms (Liu et al., 2008). There is plenty of evidence that different 5-HT receptors, such as 5-HT_3_, 5-HT_4_, 5-HT_1A_, 5-HT_2A_, and 5-HT_7_ receptors, play an important regulatory role in gastrointestinal sensation and motility (Hasler, 1999; Gershon, 2004; Tonini, 2005). The 5-HT system plays a crucial role in modulating the dysfunction of motility and sensation in the gut and is an important drug target for patients with FD.

Therefore, this study aimed to evaluate the pharmacological effects of ZZKZ on gastric hypersensitivity and motor dysfunction in patients with FD, particularly the actions of the 5-HT system in this process. Herein, functional dyspepsia was established by gastric irritation with 0.1% iodoacetamide in the neonatal period in rats (Liu et al., 2008; Cheung et al., 2013). *Ex vivo* gastric smooth muscle activity recordings and greater splanchnic afferent firing recordings were mainly employed to assess gastric motility and sensation. These results provide valuable insights into the therapeutic mechanisms of ZZKZ in patients with FD.

## 2 Materials and methods

### 2.1 Herb materials of ZZKZ

ZZKZ capsules (drug approval number: Z20020003; product batch numbers: 141042, 150106, 150422, 150838, 150840, 151255, 160401, 160402, 160403, and 160504) were provided by ShuangRen Pharmaceuticals Co. Ltd., Lonch Group, China. The dry weight of each of the following raw ingredients per 4.3 g of ZZKZ product is shown in Table 1, including *Citrus × Aurantium* L [Rutaceae; *Aurantii Fructus Immaturus*] (3.00 g), *Atractylodes Macrocephala* Koidz [Compositae; *Rhizoma Atractylodis Macrocephalae*] (4.50 g), *Bupleurum Chinense* DC [Apiaceae; *Radix Bupleuri Chinensis*] (2.25 g), and *Crataegus Pinnatifida* Bunge [Rosaceae; *Fructus Crataegi Pinnatifidae*] (2.25 g). The scientific names of plants were presented in the standard nomenclature (Rivera et al., 2014), and validated in the databases of “Plant of the World Online” (http://www.plantsoftheworldonline.org) and “The World Flora Online” (WFO, http://www.worldfloraonline.org/).

**TABLE 1:** Composition of ZZKZ capsule.

Chinese name	Scientific name^a^	Family	Drug name	Quantity (dry, g)^b^
Zhi Shi	*Citrus× Aurantium* L	Rutaceae	*Aurantii Fructus Immaturus*	3.00
Bai Zhu	*Atractylodes Macrocephala* Koidz	Compositae	*Rhizoma Atractylodis Macrocephalae*	4.50
Chai Hu	*Bupleurum Chinense *DC.	Apiaceae	*Radix Bupleuri Chinensis*	2.25
Shan Zha	*Crataegus Pinnatifida* Bunge	Rosaceae	*Fructus Crataegi Pinnatifidae*	2.25

^a^ The Latin names of plants were checked and validated in the databases of “Plant of the World Online” (http://www.plantsoftheworldonline.org) and “The World Flora Online” (WFO, http://www.worldfloraonline.org/).

^b^ The dry weight of each raw ingredient in per 4.3 g of ZZKZ, products.

### 2.2 Fingerprint of ZZKZ by HPLC analysis

This research was conducted according to the ConPhyMP guidelines (Heinrich et al., 2022), to ensure reproducibility and accurate interpretations of studies using ZZKZ aqueous extract (drug extract ratio: 18.5:1). ZZKZ weighing 1 g was dissolved in 25 ml of aqueous methanol (80%, v/v), heated to reflux in a water bath for 1.5 h, and subsequently allowed to cool naturally. Subsequently, the solution was filtered twice through a 0.45-μm microporous membrane to filter out residual material, and the continued filtrate was used as the test solution. High performance liquid chromatography (HPLC) analysis for ZZKZ sample solution was performed on an Agilent 1,260 Infinity HPLC system (Agilent technologies), with a YMC-C18 column (250.0 × 4.6 mm, 5 μm) for separation. The column temperature was maintained at 30°C, the flow rate was 1.0 ml/min, the detection wavelength was set to 276 nm, and the injected sample volume was 10 µl. The mobile phase consisted of acetonitrile (A) and purified water (B), using the following gradient elution: 0–5 min, 2% A; 5–10 min, 5% A; 10–35 min, 7% A; 35–50 min, 35% A; 50–65 min, 65% A; and >65 min, 100% A. Accordingly, the fingerprints of 10 batches of ZZKZ capsule test solution were measured, and the standard spectrum of the fingerprint was subsequently plotted.

### 2.3 Preparation of serum and supernatant containing ZZKZ

To examine the direct effects of ZZKZ on the motility of gastric smooth muscle, drug-containing serum and drug-containing supernatant were prepared respectively.

Normal adult rats were administered ZZKZ (1.0 g/kg) or saline by gavage twice a day for 7 consecutive days to achieve stable blood concentration. Two hours after the last gavage, the rats were sacrificed, and whole blood from the inferior vena cava was collected. Subsequently, ZZKZ drug-containing serum and vehicle control serum were separated after centrifugation at 3000 *g* for 15 min at 4°C and stored at −80°C for further studies on its effects on the contractile activity of the gastric muscle strip.

Intact small intestinal mucosal patches from normal adult rats were prepared by carefully stripping the seromuscular layer in ice-bathed and oxygenated Krebs’ buffer. The mucosal patches were mounted on sliders with a rectangular hole (opening area, 0.25 cm^2^) in the centre. The patches covered the entire area of the hole, maintaining an effective mucosal area of 0.25 cm^2^. The sliders were subsequently installed in a U-type chamber of the Ussing Chamber System (World Precision Instruments, United States). Each side (the mucosal and serosal sides) of the U-type chamber was filled with 5 ml of Krebs’ solution, continuously oxygenated (95% O_2_ + 5% CO_2_), and maintained at 37°C. After 15 min of equilibration, the solution on the mucosal side was replaced with 2 ml of ZZKZ solution (10 mg/ml dissolved in Krebs’ solution), while the solution on the serosal side was changed with 2 ml fresh Krebs’ solution. After 1 hour of incubation, samples were taken from the serosal side, that is, the drug-containing supernatant, representing the component absorbed and secreted by the mucosa. The ZZKZ drug-containing supernatant and vehicle control supernatant were stored at −80°C for further research on their effects on the contractile activity of the gastric muscle strip.

### 2.4 Animals and FD models

An FD model with gastrointestinal dysmotility and gastric hypersensitivity was established in rats as previously described (Liu et al., 2008; Zou et al., 2020). Sprague-Dawley rats (aged 10 days, weighing 14–18 g; Experimental Animal Center, Tongji Medical College, HUST, Wuhan, China) were used. Ten-day-old neonatal rats received 0.1% iodoacetamide (IA, soluble in 2% sucrose solution) at dose of 0.2 ml/d by gavage for 7 consecutive days to create FD models. The rats were able to develop FD-like conditions after adulthood (8 weeks of age). The rats were administered 2% sucrose solution at 0.2 ml/d for 7 days and considered as healthy controls (HC). All rats were housed under specific pathogen-free conditions at 23°C with a 12/12-h light/dark cycle and free access to food and water. The rats were weighed daily in the first week, and then their body weights were recorded weekly. At the 7^th^ week, the FD rats were treated with ZZKZ at a low (ZZKZ-L, 0.5 g/kg), medium (ZZKZ-M, 1.0 g/kg), or high dose (ZZKZ-H, 1.5 g/kg) by gavage for 7 days, while rats administered domperidone (DPLT, 3 mg/kg) were considered as treatment controls. At the 8^th^ week, the growth rate of body weight was calculated, the 3-h food intake (overnight fasting rats were allowed free access to food and water for 3 h) and 24-h food intake were recorded, and the rats were finally euthanized for further detection.

All animal experiments were approved by the Institutional Animal Care and Use Committee of Tongji Medical College, HUST, Wuhan, China. All efforts were made to minimise animal suffering and reduce the number of animals used.

### 2.5 Gastric emptying and intestine propulsion test

The gastric emptying of a solid meal was assessed in rats. The rats were fasted overnight, fed separately, allowed free access to food and water for 3 h. The 3-h food intake was subsequently calculated. Food and water were removed thereafter, and the gastric emptying of the ingested meal was assessed 3 h later. Finally, the stomach was removed after the rats were euthanized and weighed before and after thorough emptying, and the D-value was calculated as the gastric food residue. Therefore, the formula for gastric emptying was as follows: gastric emptying (%) = 100 − (gastric food residue/food intake) × 100.

Gastric emptying and intestinal propulsion were further assessed using phenol red meal. The rats were fasted overnight and subsequently gavaged with 2 ml of 10% hydroxymethyl cellulose containing 0.04% phenol red (original solution). The rats were anaesthetised and sacrificed 30 min later, and their stomachs and small intestines were immediately removed. For the assessment of gastric emptying, the stomach was cut open along the greater curvature and fully washed off in 30 ml of 0.5 mol/L NaOH solution. Thereafter, the washing solution was centrifuged (3000 repetitions, 10 min), and the supernatant was obtained and detected colorimetrically using a spectrophotometer at 560 nm. Therefore, the residual rate of phenol red was calculated as follows: residual rate of phenol red (%) = OD_560_ of washing solution/OD_560_ of the original solution × 100. To measure intestinal propulsion, the small intestine was laid out on white paper, and the distance of phenol red in the small intestine and total length of the small intestine were measured. Thus, the mall intestinal propulsion rate was calculated as follows: small intestinal propulsion rate (%) = distance of phenol red in the small intestine/total length of the small intestine × 100.

### 2.6 Gastric smooth muscle activity recording

The activity of the gastric smooth muscle was measured by an *ex vivo* recording of isometric contractions of the longitudinal muscle strips. Briefly, after stripping the mucosal layer, the muscle strips (10 mm in length and 3 mm in width) were prepared along the longitudinal axes of the tract. Thereafter, muscle strips were bathed in individual 10-ml chambers filled with oxygenated (95% O_2_ + 5% CO_2_) Krebs’ solution (119 mM NaCl, 4.7 mM KCl, 1.2 mM NaH_2_PO_4_, 25 mM NaHCO_3_, 2.5 mM CaCl_2_, 1.2 mM MgSO_4_, and 11.1 mM glucose; pH 7.30–7.40) and maintained with constant temperature at 37°C on an organ bath system. One end of the strip was fixed with thin wires, and the other end was connected to a TRI201AD Isometric Transducer (AD Instruments, Australia), which was connected to a Quad Bridge Signal Amplifier (AD Instruments, Australia) and PowerLab 8/35 analog-digital converter (AD Instruments, Australia). A preload of 1.5 g was applied on each strip, and the contractile curve was consecutively recorded and analysed using LabChart software version 7.0 (AD Instruments, Australia). The strip was equilibrated for 30 min to achieve stable spontaneous contractions. Spontaneous smooth muscle activity was recorded, and the frequency, amplitude, and motility index (MI) of spontaneous contractions were calculated. MI is defined as the area under the contractile curve (AUC) per unit time, reflecting the comprehensive contractility of smooth muscle strips, including tension, amplitude, and frequency (Zhang et al., 2019). To evaluate the nitrergic and cholinergic responses of strips, NG-nitro-L-arginine methyl ester (L-NAME, 10^−5^ mol/L) and different concentrations of acetylcholine (ACh, 10^−8^ mol/L, 10^−7^ mol/L, 10^−6^ mol/L, 10^−5^ mol/L, and 10^−4^ mol/L) were successively added to the bath chamber. Contractile curves were recorded, and the half-effective concentration (EC_50_) of ACh was calculated. To assess the response to 5-HT in strips, 5-HT (0.1 mM) was added to the bath chamber, and the contractile response was recorded and analysed. The contractile response induced by 80 mM K^+^ (high K^+^-mediated depolarisation) is considered a reference for the maximum contractility of muscle strips.

### 2.7 Greater splanchnic afferent activity recording

The rats were anaesthetised with intraperitoneal sodium pentobarbital at a dose of 50 mg/kg. The stomach with the attached mesentery and tissues was gently removed and used for recording immediately after the rats were euthanised. The stomach was bathed in the outer pool of a recording chamber with circulating oxygenated (95% O_2_ + 5% CO_2_) Krebs ' solution at 37°C. The proximal end (cardia) of the stomach was attached to an input tube connected to a syringe pump, whereas the distal end (pylorus) was attached to an output tube with a three-way valve connected to a manometer. The left greater splanchnic nerve (GSN) was carefully separated under a dissecting microscope and placed in the inner pool of the recording chamber through a small hole between the inner and outer baths after which the hole was blocked with vaseline. The GSN was draped over a recording electrode using a micro manipulator, and an equally fine bundle of connective tissue was suspended on the reference electrode. The inner pool was immersed in warm paraffin oil to prevent the nerves from drying. GSN discharge was recorded using a Model-1800 Microelectrode AC Amplifier (A-M Systems, United States) and a PowerLab 4/26 data acquisition system (AD Instruments, Australia) with filtering (bandpass, 10–10000 Hz) and acquisition (20 KHz sampling rate). Spontaneous firing of the GSN was recorded after 1 h of stabilisation. The discharge activity of the GSN in response to a short test distention stimulus (60 mmHg) increased by 30% over baseline and was considered a responsive fibre. To test the responses of the GSN afferent nerves to chemical or mechanical stimuli, perfusion with HCl (5 mM) or gastric distention (intragastric pressure of 20 mmHg for low-threshold fibres and 60 mmHg for high-threshold fibres) in the stomach was performed. Each distention was recorded for at least 30 s at 5-min intervals. Moreover, the response to 5-HT (0.1 mM) and effect of the 5-HT_3_ receptor antagonist GR-68755 (alosetron, 1 μM) were further recorded in GVNs.

### 2.8 Enzyme linked immunosorbent assay

The whole blood of rats was collected, and the serum was separated after centrifugation at 3000 *g* for 15 min at 4°C. The samples were stored at −80°C for further analysis. Serum 5-HT levels were measured using a Serotonin Enzyme Linked Immunosorbent Assay (ELISA) Kit (ARG80480, Arigo Biolaboratories, China) according to the manufacturer’s instructions.

### 2.9 Real-time quantitative PCR analysis

Total RNA from gastric tissues was extracted using a Trizol Reagent (Invitrogen, Life Technologies). A two-step real-time quantitative PCR was subsequently performed. Briefly, a PrimeScript^TM^ RT Master Mix Kit (TaKaRa) was used to synthesise cDNA and a QuantiTest SYBR Green PCR Kit (QIAGEN) was used for RT-qPCR on a ROCHE LightCycler ^®^ 480 System according to the manufacturer’s instructions. The primer sequences were as follows: TPH1 (forward 5′-CAA​GGA​GAA​CAA​AGA​CCA​TTC-3′ and reserve 5′-ATT​CAG​CTG​TTC​TCG​GTT​GAT​G-3′); SERT (forward 5′-TCCGCA TGAATGCTGTGTAAC-3′ and reserve 5′-TTG​GCT​TAG​AGG​GGA​GGA​GTC-3′); 5-HT1A (forward 5′-CGT​GCA​CCA​TCA​GCA​AGG​A-3′ and reserve 5′-CTGAAGATGC GCCCGTAGAGA-3′); 5-HT2A (forward 5′-ACC​GCT​ATG​TCG​CCA​TCC​A-3′ and reserve 5′-GAC​CTT​CGA​ATC​ATC​CTG​TAG​TCC​A-3′); 5-HT3 (forward 5′-CGCCTG TAGCCTTGACATCTAT-3′ and reserve 5′-CGA​CCT​CAC​TTC​TTC​TGG​TGT​T-3′); 5-HT4 (forward 5′-GGG​AGA​TGT​TTT​GCC​TGG​TC-3′ and reserve 5′-CGATGTGTG CTGTGCTGGTC-3′); 5-HT7 (forward 5′-GCT​CAT​CAC​GCT​GCT​GAC​GAT-3′ and reserve 5′-CGC​CAG​GGA​CAC​AAT​CAG​G-3′); and GAPDH (forward 5′-ACCACAGTC CATGCCATCAC-3′ and reserve 5′-TCC​ACC​ACC​CTG​TTG​CTG​TA-3′). Dissociation curves were plotted to confirm single amplification. Endogenous GAPDH was used as a normalisation reference. The relative expression of the mRNA species was quantified using the 2^−ΔΔCT^ method.

### 2.10 Data expression and statistical analysis

All data are presented as mean ± standard error (SEM). Paired or unpaired Student’s t-tests or one-way analysis of variance (ANOVA) was used for the comparison of data where applicable. A *p*-value < 0.05 indicated statistical significance.

## 3 Results

### 3.1 Fingerprint of ZZKZ

**FIGURE 1:**
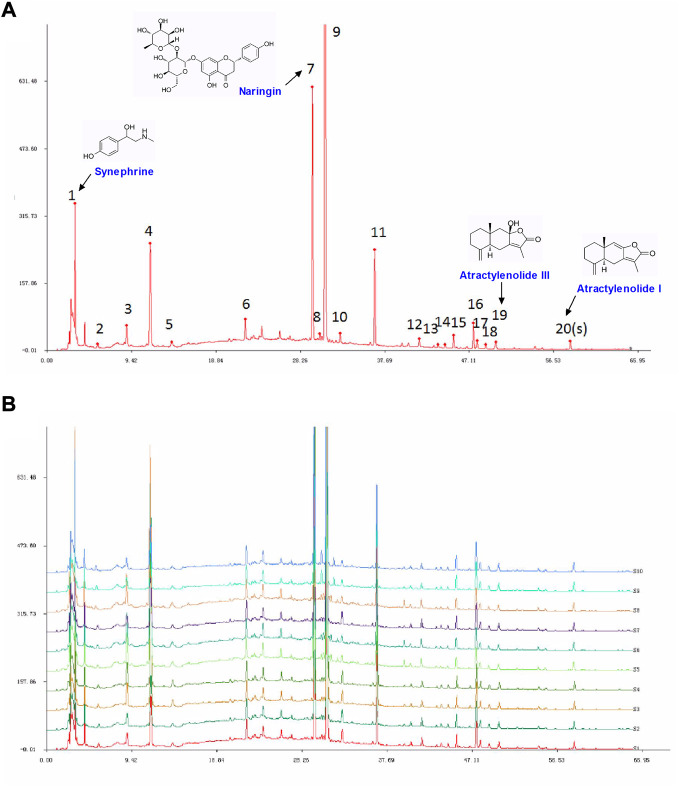
HLPC fingerprint of the standard reference and ZZKZ. **(A)** Chromatogram of standard references is detected at 276 nm. **(B)** Reproducible HPLC chromatograms of ZZKZ from 10 batches. (1) synephrine, (7) naringin, (19) atractylenolide III, and (20) atractylenolide I.

The HPLC fingerprints of the standard reference and ZZKZ are shown in Figure 1. The similarity evaluation results showed that the similarity of 10 batches of samples was 0.998 or 0.999. Twenty characteristic peaks were detected at 276 nm which were attributed to the four botanical drugs in ZZKZ. Some peaks can be attributed to more than one herb, while the most common peaks were derived from the immature fruit of *Citrus × Aurantium* L., meaning that it plays an important role in whole party components (Supplementary Table S1). Furthermore, four peaks were preliminarily identified by comparison with corresponding reference standards, including synephrine (1), naringin (7), atractylenolide III (19), and atractylenolide I (20).

### 3.2 ZZKZ modulated the contractile activity of gastric smooth muscle *ex vivo*

**FIGURE 2:**
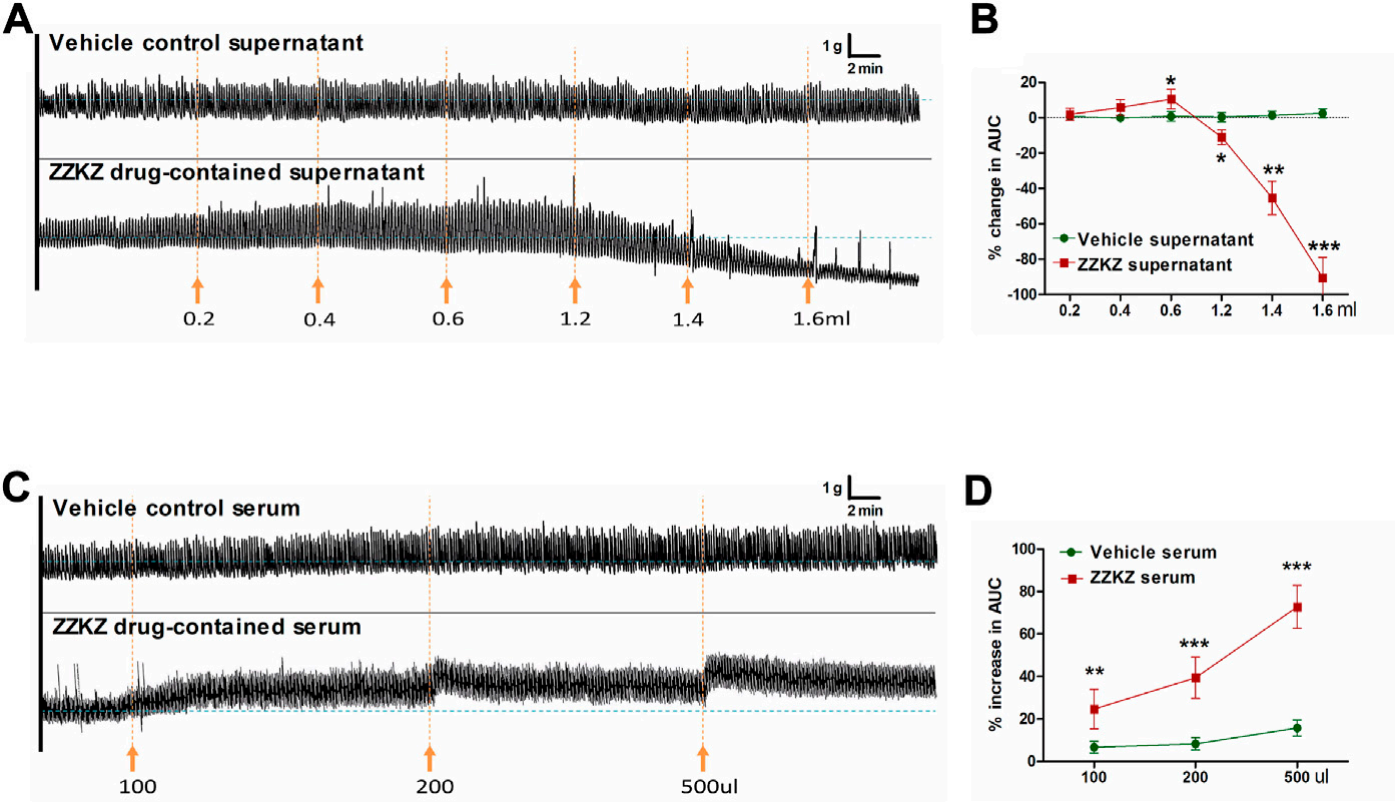
ZZKZ modulated the contractile activity of gastric smooth muscle *ex vivo*. **(A,B)** Effects of ZZKZ drug-contained supernatant on gastric smooth muscle strips. **(C,D)** Effects of ZZKZ drug-contained serum on gastric smooth muscle strips. N = 8; *p < 0.05, **p < 0.01, ***p < 0.001 vs*.* vehicle control.

The effects of ZZKZ on the motility of *ex vivo* gastric smooth muscle strips were evaluated, and the drug-containing serum and drug-containing supernatant of ZZKZ were used. The drug-containing supernatant contained mainly the ingredients of ZZKZ absorbed by the intestinal mucosa, indicating the direct action of ZZKZ on gastric motility. The ZZKZ drug-contained supernatant showed a bidirectional regulation on the contraction of gastric smooth muscle strips, with a mild facilitative effect at low doses (0.2–0.6 ml) and a significant inhibitory action at higher doses (1.2–1.6 ml) (Figures 2A,B). In addition, drug-containing serum is a mixture of absorbed drugs and their metabolites in the blood, as well as endogenous products induced by the drugs after acting in the body, reflecting the *in vivo* effect of ZZKZ when reaching a stable blood concentration. The ZZKZ drug-containing serum, but not the vehicle control serum, dose-dependently promoted the contractions of gastric smooth muscle strips (Figures 2C,D).

### 3.3 ZZKZ promoted the weight gain, food intake, gastric emptying, and intestinal propulsion in FD rats

**FIGURE 3:**
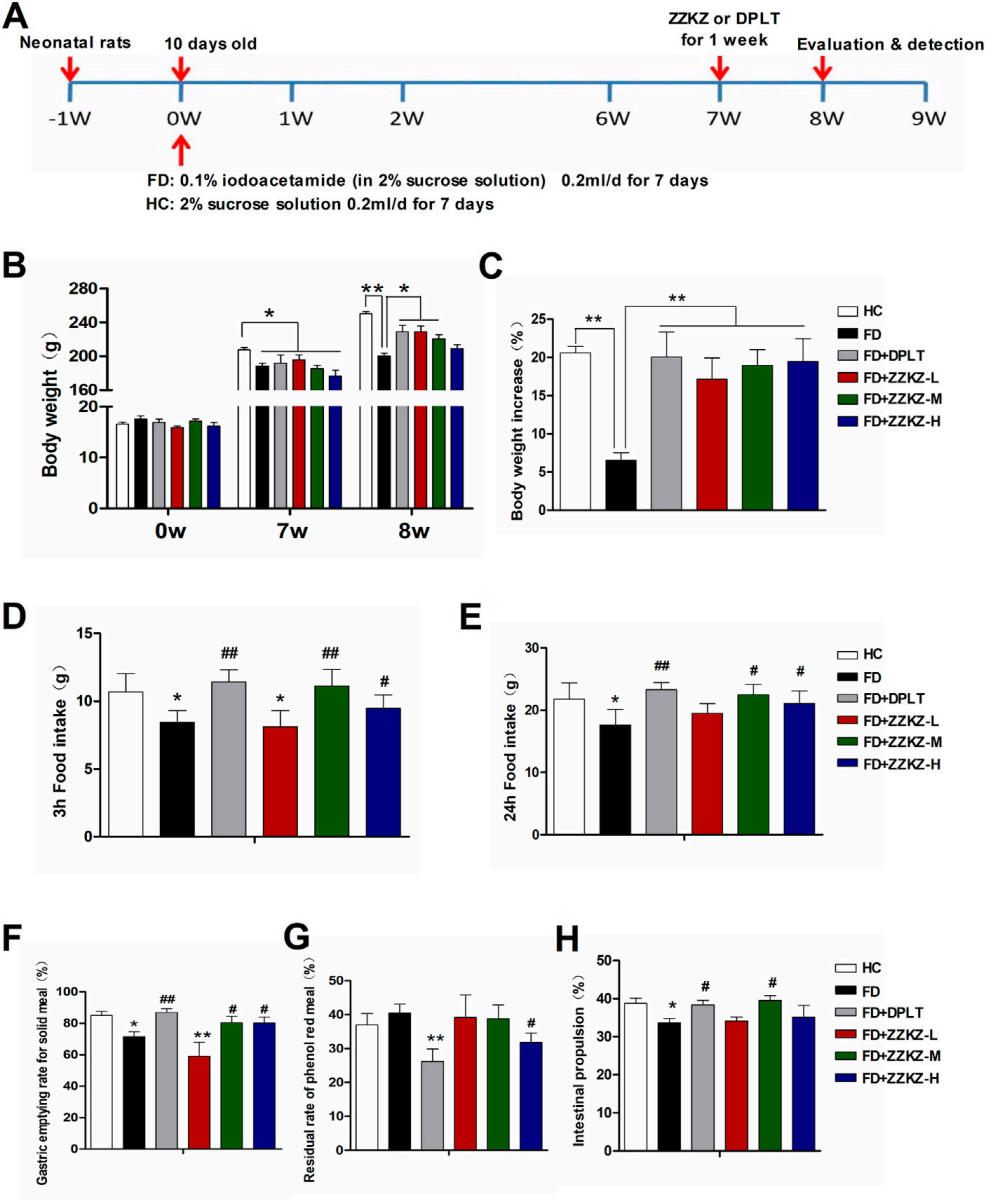
ZZKZ promoted weight gain, food intake, gastric emptying and intestinal propulsion in FD rats. **(A)** The study protocol of FD models and treatment. **(B)** Changes in body weight, and **(C)** body weight gain in the week after ZZKZ treatment in different groups. N = 6–8; *p < 0.05, **p < 0.01 **(D–E)** Food intake in different groups, including quick eating (3-h food intake) and total ingestion (24-h food intake). N = 6–8; *p < 0.05 vs*.* HC; ^#^ *p* < 0.05, ^##^ *p* < 0.01 vs*.* FD. **(F)** Gastric emptying rate for the solid meal, **(G)** residual rate of phenol red meal, and **(H)** intestinal propulsion rate in different groups. N = 6–8; *p < 0.05, **p < 0.01 vs*.* HC; ^#^ *p* < 0.05, ^##^ *p* < 0.01 vs*.* FD.

Adult IA-induced FD rats had a lower body weight than normal controls. There was a significant weight gain in FD rats treated with ZZKZ and domperidone (Figures 3B,C). Food intake was obviously inhibited in FD rats, including quick eating (3 h food intake) and total ingestion (24 h food intake), yet these were markedly improved by domperidone, ZZKZ-M, and ZZKZ-H (Figures 3D,E). FD rats showed delayed gastric emptying, with a decreased gastric emptying rate for solid meals (Figure 3F) and increased residual rate of phenol red meal (Figure 3G), as well as retarded intestinal propulsion (Figure 3H). To some extent, domperidone and ZZKZ effectively improved gastric motility by restoring gastric emptying and intestinal propulsion in FD rats (Figures 3F–H).

### 3.4 ZZKZ promoted the spontaneous and ACh-induced contractions of gastric smooth muscle in FD rats

**FIGURE 4:**
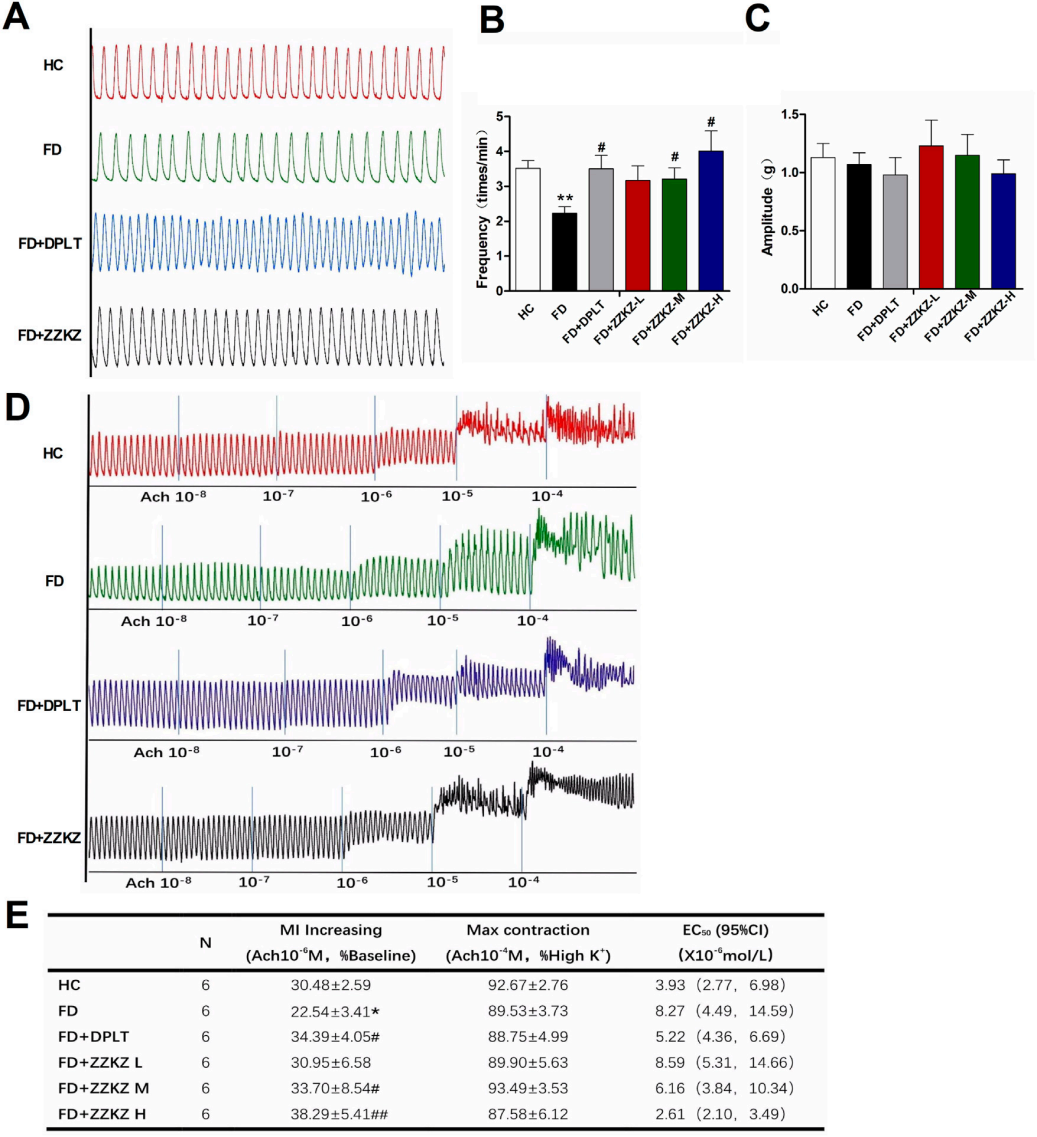
ZZKZ promoted the contractions of gastric smooth muscle in FD rats. **(A)** The typical spontaneous contractile curve of gastric smooth muscle strips. **(B,C)** Frequency and amplitude of spontaneous contractions in different groups. N = 6; **p < 0.01 vs*.* HC; ^#^ *p* < 0.05 vs*.* FD **(D)** The typical contractile curve of gastric muscle strips to gradient acetylcholine (10^−8^ mol/L to10^−4^ mol/L) **(E)** Statistics of increasing in MI induced by ACh at a dose of 10^−6^ mol/L, max contraction induced by ACh at a dose of 10^−4^ mol/L, and concentration at 50% of maximal effect (EC_50_) of ACh to the contractions of gastric muscle strips in different groups. N = 6; *p < 0.05 vs*.* HC; ^#^ *p* < 0.05, ^##^ *p* < 0.01 vs*.* FD.

The spontaneous contractile activity of gastric smooth muscle strips was decreased in FD rats (Figure 4A), with decreased frequency, but not amplitude (Figures 4B,C). In particular, the frequency of spontaneous gastric contractions was improved by treatment with domperidone, ZZKZ-M, or ZZKZ-H in FD rats (Figures 4A,B). The contractile response of the gastric muscle strips to gradient ACh was further evaluated in ZZKZ-treated or untreated FD rats. It indicated that the gastric muscle strips were less responsive to ACh in FD rats relative to HC, with a lower amount of MI increases induced by ACh 10^−6^ M (% baseline, 22.54 ± 3.41 vs. 30.48 ± 2.59; *p* < 0.05), but not the max contraction induced by ACh 10^−4^ M (% high K^+^, 89.53.54 ± 3.73 vs. 92.67 ± 2.76; *p* > 0.05) (Figures 4D,E). Meanwhile, the EC_50_ of ACh to contractions of gastric muscle strips increased in FD rats (Figure 4E). The MI increasing induced by ACh 10^−6^ M was enlarged with a relatively low EC_50_ of ACh to the gastric muscle strips in FD rats by domperidone, ZZKZ-M, or ZZKZ-H (Figures 4D,E).

### 3.5 ZZKZ alleviated spontaneous activity, and the chemical and mechanical stimulated firing of greater splanchnic afferents in FD rats

**FIGURE 5:**
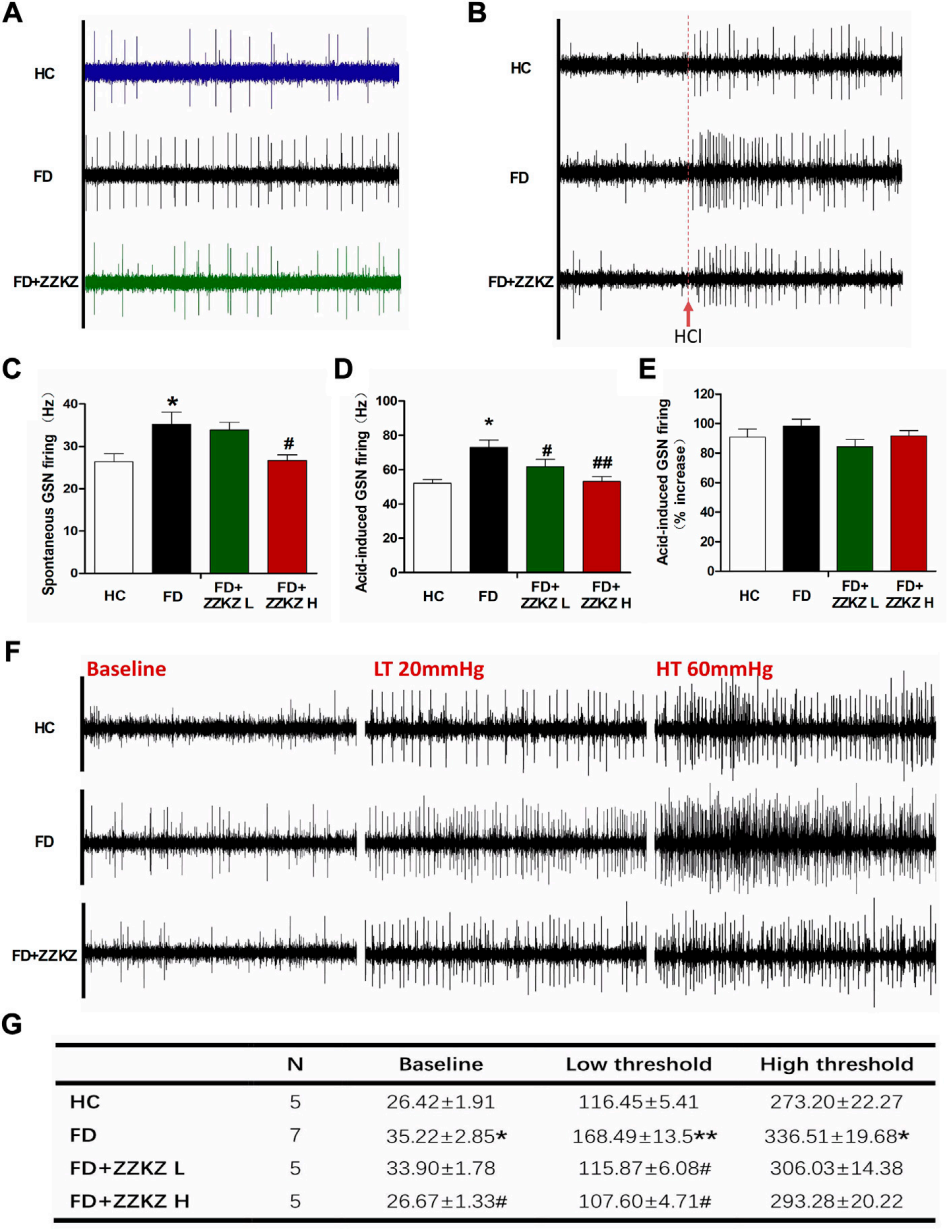
ZZKZ alleviated the spontaneous and stimulated firing of greater splanchnic afferents in FD rats. **(A)** Typical records of spontaneous GSN firing in different groups. **(B)** Frequency of spontaneous GSN discharge. N = 6; *p < 0.05 vs*.* HC; ^#^ *p* < 0.05 vs*.* FD. **(C)** The typical records of acid (5 mM HCl)-induced GSN firing. **(D,E)** Frequency of acid-induced GSN discharge. N = 6; *p < 0.05 vs*.* HC; ^##^ *p* < 0.01 vs*.* FD. **(F)** The typical records of GSN firing induced by intragastric distension, including the low threshold fibres (20 mmHg) and high threshold fibres (60 mmHg). **(G)** Statistics of the frequency GSN firing at baseline, and at the conditions of 20 mmHg and 60 mmHg intragastric distension. N = 5–7; *p < 0.05, **p < 0.01 vs*.* HC; ^#^ *p* < 0.05 vs*.* FD.

The spontaneous activity of the GSN was higher in FD rats than in healthy controls, which is a feature of gastric sensory hypersensitivity. ZZKZ-H significantly reduced the spontaneous firing frequency of GSN in FD rats (Figures 5A,B). Acid (5 mM of HCl) induced apparent GSN firing, which was significantly enhanced with firing frequency in FD rats. The latter could also be improved by ZZKZ-H (Figures 5C,D). Subsequently, mechanically stimulated GSN firing was tested by increasing the intragastric pressure, including low-threshold fibres (20 mmHg) and high-threshold fibres (60 mmHg). FD rats were more sensitive to intragastric distension with increased firing responses to both 20 mmHg and 60 mmHg stimuli (Figures 5F,G). For the low threshold fibres of GSN, ZZKZ markedly reduced the firing response to the stimulus of intragastric distension in FD rats. However, the firing frequency of high-threshold fibres of GSN was not significantly alleviated by ZZKZ in FD rats (Figures 5F,G). This indicates that ZZKZ may improve the hypersensitive response to gastric distention in FD rats, especially for low-threshold afferents.

### 3.6 ZZKZ ameliorated gastric smooth muscle contraction and greater splanchnic afferent firing induced by 5-HT in FD rats

**FIGURE 6:**
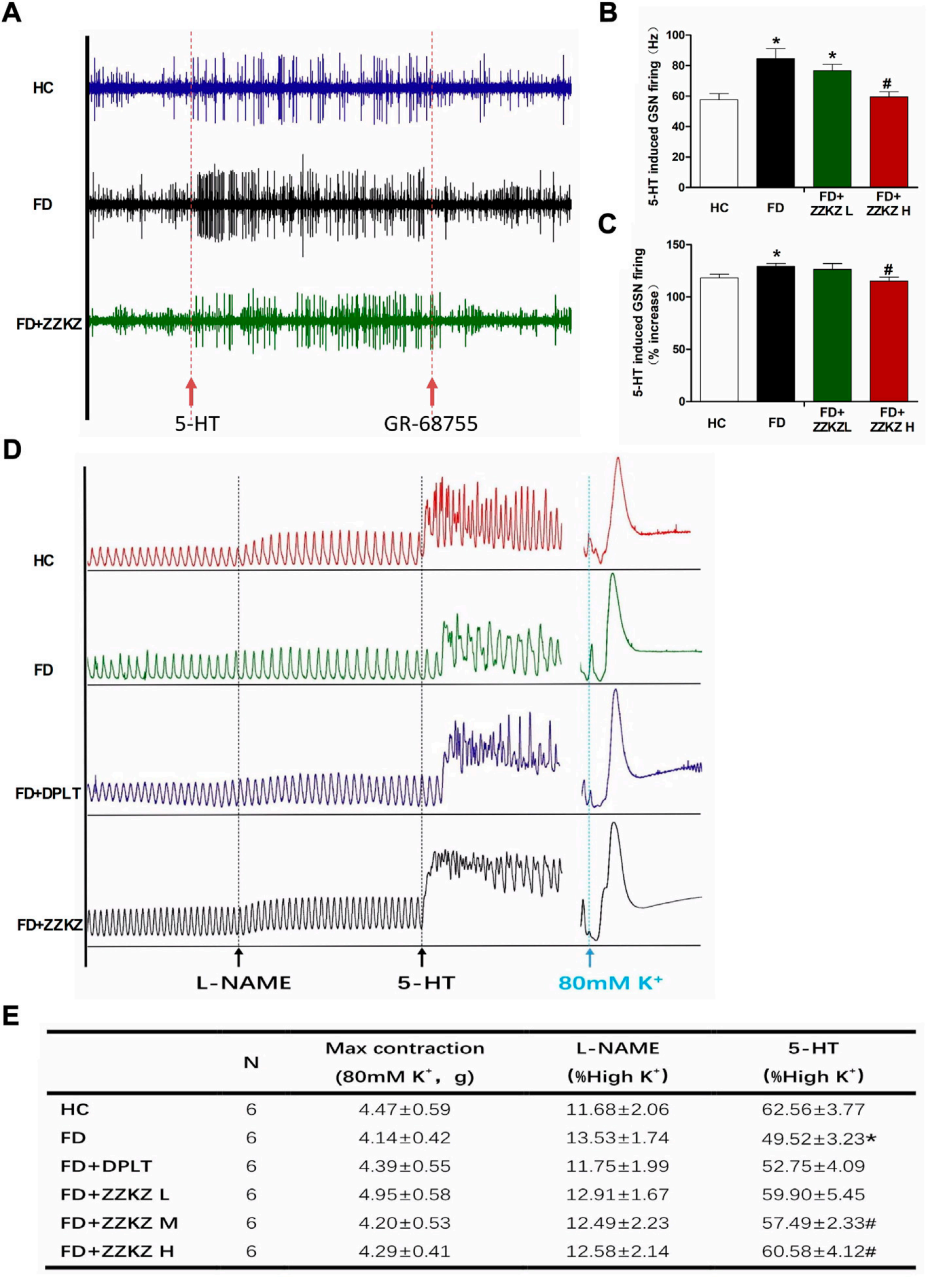
ZZKZ ameliorated 5-HT-induced greater splanchnic afferents firing and smooth muscle contraction in FD rats. **(A)** The typical records of 5-HT induced GSN firing, as well as the effects of GR-68755. **(B,C)** Frequency of 5-HT-induced GSN discharge. N = 6; *p < 0.05 vs*.* HC; ^#^ *p* < 0.05 vs*.* FD **(D)** The typical contractile curve of gastric smooth muscle strips induced by L-NAME and 5-HT. The contractile response induced by 80 mM K+ (high K + -mediated depolarisation) is considered as a reference for maximum contractility of the muscle strips. **(E)** Statistics of the motility index (MI) of gastric muscle strips, including L-NAME and 5-HT induced contractions, and the max contraction in different groups. N = 6; *p < 0.05 vs*.* HC; ^#^ *p* < 0.05 vs*.* FD.

The 5-HT pathway may play a key role in gastric dysmotility and visceral hypersensitivity in FD rats, and in the mechanism of ZZKZ. 5-HT induced apparent GSN firing, which was more intense in FD rats and may be blocked by GR-68755, a 5-HT_3_ receptor antagonist (Figure 6A). The GSN firing induced by 5-HT was effectively inhibited by ZZKZ treatment in FD rats (Figures 6B,C). Moreover, Figures 6B,Cthe gastric muscle strips of FD rats exhibited a lower response to 5-HT under NO blocking by L-NAME compared with healthy controls (Figures 6D,E). The hypomotility of gastric muscle strips in FD rats was markedly improved by treatment with domperidone and ZZKZ, especially in the medium-to high-dose groups (Figures 6D,E).

### 3.7 ZZKZ promoted the release of serum 5-HT and regulated the gastric expression of 5-HT receptors in FD rats

**FIGURE 7:**
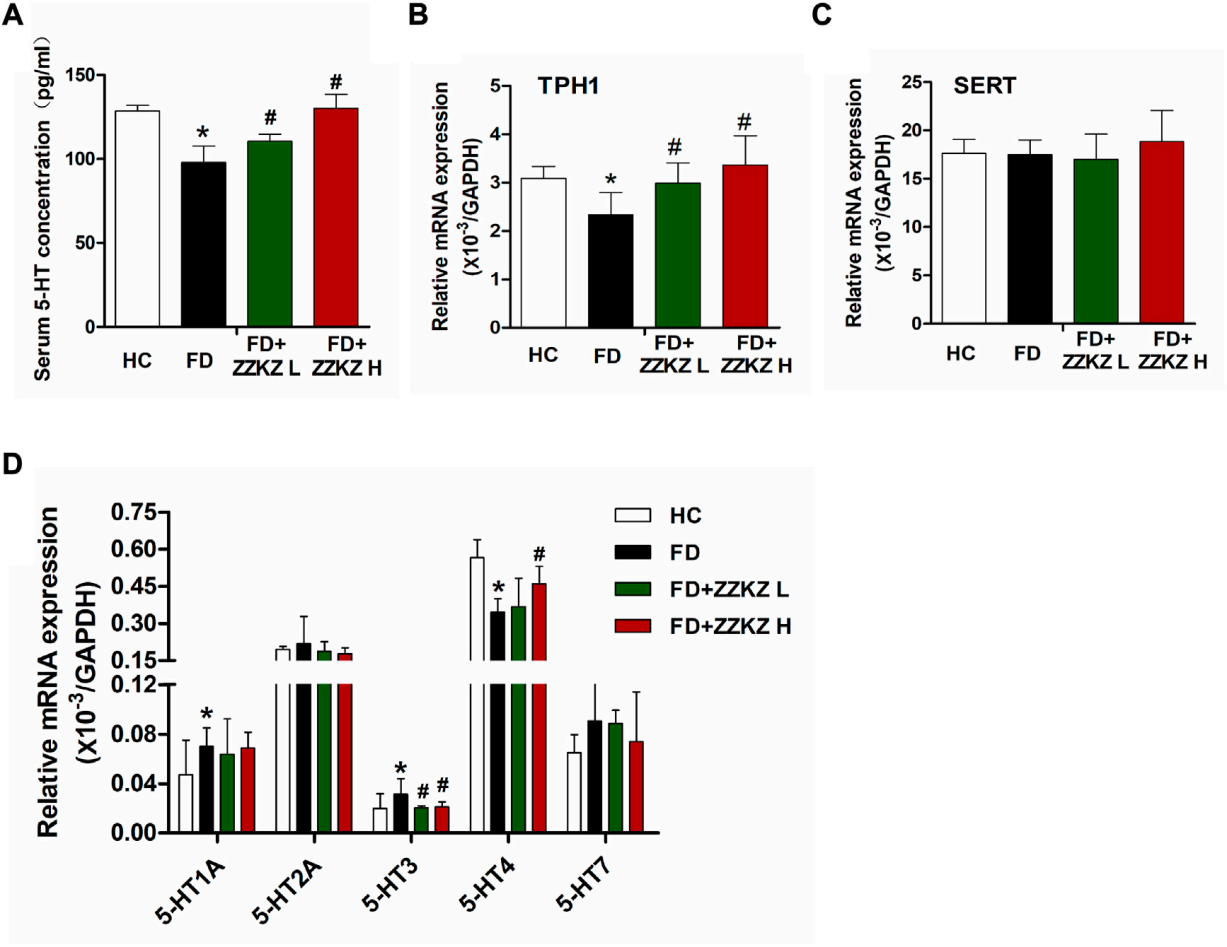
ZZKZ regulated the levels of serum 5-HT and its receptors in the gut of FD rats. **(A)** Concentration of 5-HT in serum. **(B,C)** The mRNA expression of tryptophan hydroxylase 1 (TPH1) and serotonin transporter (SERT) in gastric tissues. **(D)** The gene expression of gastric 5-HT receptors, including 5-HT_1A_, 5-HT_2A_, 5-HT_3_, 5-HT_4_, 5-HT_7_ receptors. N = 6–8; *p < 0.05 vs*.* HC; ^#^ *p* < 0.05 vs*.* FD.

Low levels of serum 5-HT and gastric TPH1 were observed in FD rats, which may be improved in ZZKZ-treated FD rats (Figures 7A,B). Gene expression of SERT in gastric tissues was not significantly changed in FD rats (Figure 7C). However, the mRNA levels of 5-HT receptors, including 5-HT_1A_ and 5-HT_3_, were upregulated in FD rats, while the expression of 5-HT_4_ receptors was decreased in FD rats (Figure 7D). Notably, ZZKZ inhibited the gene expression of 5-HT_3_ receptors in FD rats and increased the expression of 5-HT_4_ receptors in the gut of FD rats (Figure 7D). This further indicated that the 5-HT system is involved in the mechanism for improving gastric dysmotility and hypersensitivity in FD rats by ZZKZ.

## 4 Discussion

Gastric hypersensitivity and motor dysfunction are believed to be two of the most important pathophysiologies of FD symptoms (Camilleri et al., 2016; Tack et al., 2018; Miwa et al., 2022). A previous study has shown that ZZKZ effectively improves postprandial fullness, early satiety, epigastric burning, and total symptom scores in patients with FD (Xiao et al., 2019), although the exact mechanisms remain to be established. In the current study, we found that ZZKZ promoted food intake, gastric emptying, and intestinal propulsion, as well as improved the contractions of gastric smooth muscle strips in FD. ZZKZ alleviated greater splanchnic afferent firing in response to intragastric distension and acid perfusion in FD rats. Herein, ZZKZ improved FD-related gastric hypomotility and hypersensitivity and may be an ancient but promising compound for relieving FD symptoms.

First, we tested the *ex vivo* actions of ZZKZ on the contractions of gastric smooth muscle strips. Results have shown that ZZKZ drug-containing serum significantly promoted the contractions of gastric smooth muscle strips, while the direct effects of ZZKZ on gastric smooth muscle identified by drug-containing supernatants were primarily inhibitory. Therefore, ZZKZ is unlikely to act directly on the gastric neuromuscular system to improve gastric motility and FD symptoms but to modulate the internal environment and induce some active products in the body.

Furthermore, ZZKZ exhibited obvious *in vivo* prokinetic effects in FD rats, including accelerated gastric emptying and intestinal propulsion, and increased food intake. Treatment with ZZKZ promoted the spontaneous contractions of gastric smooth muscle and enhanced the response to ACh in FD rats, indicating improved gastric hypomotility in FD rats. None of the studies have examined the mechanisms by which ZZKZ regulates gastrointestinal motility; however, some studies have reported the prokinetic actions of ZZKZ ingredients. *Fructus Aurantii* extract can enhance gastrointestinal motility by altering 5-HT and VIP expression in the stomach antrum and duodenal mucosa of rats (Jiang et al., 2014). The aqueous extracts from *Fructus Aurantii Immaturus* strengthen bowel movement in cathartic colons by increasing the expression of 5-HT_4_ receptor and neurofilament-H (Wang et al., 2015). Citrus flavanones, includning hesperidin and naringin, have the potential to improve gastrointestinal barrier function and intestinal inflammation (Stevens et al., 2019). Moreover, the crude extracts and pure compounds of *Atractylodes Macrocephala* Koidz. are often used to treat gastrointestinal hypofunction (Zhu et al., 2018; Yang et al., 2021), including spleen-deficiency and qi-stagnation in traditional Chinese medicine (Yang et al., 2021). Atractylenolide III, a class of lactone compounds derived from *Atractylodes macrocephala* Koidz., promotes distal colonic contraction by promoting the synthesis of the acetylcholinergic receptor M3 (Choi et al., 2011; Deng et al., 2021). In addition, *Crataegus Pinnatifida* Bunge (Hawthorn) has been widely used for the treatment of dyspepsia (Wu et al., 2014). The aqueous extract of *Crataegus Pinnatifida* significantly enhanced the contractility of rat gastric and intestinal smooth muscle strips in a dose-dependent manner, and a higher dosage increased the intensive contraction induced by acetylcholine and antagonised muscle relaxation induced by atropine (Wu et al., 2014). Above all, *Citrus Aurantium*, *Atractylodes Macrocephala* and *Crataegus Pinnatifida* in the ZZKZ formula are beneficial for improving gastrointestinal hypofunction, which may exert synergistic action in the treatment of FD.

Gastric hypersensitivity is also regarded as a hallmark of FD and is observed in 35%–65% of FD patients, which is characterised by an increased perception of gastric mechanical distension and acid irritation (Tack et al., 2001; Vanheel and Farre, 2013). The afferent sensitization has been suggested in the process of visceral hypersensitivity (Deiteren et al., 2015). We examined the activity of spinal afferents travelling in the GSN, which are first-order neurones that respond to noxious stimuli in the stomach. Spontaneous firing and induced activity by chemical and mechanical stimulation in GSN afferents were significantly increased in FD rats compared with controls, which was consistent with the results observed previously (Samsom et al., 1999; Liu et al., 2008; Page and Li, 2018).

We found that ZZKZ inhibited GSN afferent firing in response to acid perfusion and intragastric distension in FD rats. It has been reported that the hyper-response of gastric afferent fibres to acid irritation may be correlated with epigastric burning in patients with FD (Talley, 2017). Moreover, gastric distension increases activity in brain areas that regulate eating habits and pain in patients with FD (Vandenberghe et al., 2007). Visceral hypersensitivity to gastric distension appears to be correlated with postprandial pain (Di Stefano et al., 2014). Other nonpainful symptoms, including fullness, satiety, and nausea, are also triggered at lower intense distention in the sensitised stomach (Vandenberghe et al., 2005; Liu et al., 2008). Further studies have indicated that ZZKZ markedly depressed the firing response of low-threshold fibres, but not of high-hreshold fibres, in the GSN to intragastric distension. As known, the low-threshold fibres, which deliver non-nociceptive signals, are involved in the regulation of satiety and food intake, as well as the reflexive regulation of motility, secretion, and absorption, whereas high-threshold fibres are largely responsible for encoding gut nociceptive signals (Blackshaw et al., 2007). FD symptoms mainly result from increased sensitivity of low-threshold fibres, leading to the dysregulation of innocuous signaling (Wang et al., 2012). Accordingly, ZZKZ may improve FD-related visceral hypersensitivity and symptoms, including gastric pain, burning, fullness, and satiety, by suppressing the activity of GSN afferents to chemical (acid irritation) and mechanical (intragastric distension) stimuli.

Additionally, further results have demonstrated that ZZKZ ameliorated the hyperactivity of GSN afferents and the hypo-contraction of gastric smooth muscle strips in FD rats, *via* mechanisms involving the gastric 5-HT system. Exogenous 5-HT stimulated the significant firing of GSN afferents, which was more intense in FD rats than in HCs. The aforementioned firing improved in ZZKZ-treated FD rats. The discharge of GSN afferents can be blocked by alosetron, a specific 5-HT_3_ receptor inhibitor. 5-HT widely participates in the regulation of gastrointestinal reflex, nausea and vomiting, as well as gastrointestinal hyperalgesia and pain, by activating 5-HT_3_ receptors on afferents (Browning, 2015). This indicates that ZZKZ may regulate the activities of sensory afferents in the GSN *via* the 5-HT and 5-HT_3_ receptor pathways in FD rats. Beyond that, treatment with ZZKZ promoted the contractile response of gastric muscle strips to 5-HT in FD rats. 5-HT accelerates gastric peristalsis by activating presynaptic 5-HT_4_ receptors which mediate acetylcholine release (Kendig and Grider, 2015; Keating and Spencer, 2019). Thus, 5-HT_4_ receptors play a key role in the ZZKZ-induced amelioration of gastrointestinal motility in FD rats.

We also found that ZZKZ increased the gene expression of gastric TPH1 and increased the levels of serum 5-HT in FD rats. Notably, ZZKZ treatment inhibited the gene expression of 5-HT_3_ receptors in FD rats and may potentially increase the levels of 5-HT_4_ receptors in the gut of FD rats. Research showed that *Aurantii Fructus Immaturus* significantly increased 5-HT-positive fibres in the stomach antrum, duodenal mucosa, and jejunal mucosa of rats (Jiang et al., 2014). It can also increase the levels of serum 5-HT (Jiang et al., 2014) and up-regulate the expression of 5-HT_4_ receptors in the gut (Wang et al., 2015). Evidence has also demonstrated that *Atractylodis Macrocephalae Rhizoma* could inhibit the activation of the 5-HT_3_ receptor (Kimura and Sumiyoshi, 2012), and its active ingredient, Atractylenolide III, could promote the release of 5-HT (Deng et al., 2021). These results further suggested that the ZZKZ compound may modulate 5-HT signals to improve gut motility and feelings in patients with FD.

he current study firstly provided insight into the multiple effects and mechanisms of ZZKZ compounds in improving FD symptoms. However, several potential limitations still need to be explained. First, we used a series dosages of ZZKZ to assess the pharmacological effects and its dose correlation, including the equivalent dose (1.0 g/kg/day) in clinical use matrixing *via* body surface area (Reagan-Shaw et al., 2008), as well as a lower dose (0.5 g/kg/day) and a higher dose (1.5 g/kg/day). More reasonable doses setting is necessary in the following studies, since excessive dosage will presumably result in artefacts and may exceed the pharmacologically meaningful level. Secondly, we assessed the therapeutic effects of multicomponent ZZKZ extracts, however, the specific active substances really works in improving FD symptoms need to be further validated.

## 5 Conclusion

In conclusion, ZZKZ improves FD-related gastric hypersensitivity and motor dysfunction, and the gastric 5-HT system with lower 5-HT_3_ activity and increased 5-HT_4_ distribution was involved in the mechanisms of ZZKZ in treating FD. These results provide a basis for the clinical application of ZZKZ, which is an effective compound for relieving FD symptoms, and further investigations to explore the underlying mechanisms of ZZKZ at the molecular level.

## Data Availability

The original contributions presented in the study are included in the article/Supplementary Material, further inquiries can be directed to the corresponding authors.

## Abbreviations

ACh, acetylcholine; DPLT, domperidone; EC50, concentration for 50% of maximal effect; L-NAME, NG-nitro-L-arginine methyl ester; GSN, greater splanchnic afferent nerve; LT, low threshold; HT, high threshold; 5-HT, 5-Hydroxytryptamine; FD, functional dyspepsia; HC, healthy control; HPLC, high performance liquid chromatography; IA, iodoacetamide; MI, motility index; TPH1, tryptophan hydroxylase 1; SERT, serotonin transporter; TCM, traditional Chinese medicine; ZZKZ, Zhizhu Kuanzhong capsule.
