# Supplementary material for: Zhizhu Kuanzhong, a traditional Chinese medicine, alleviates gastric hypersensitivity and motor dysfunction on a rat model of functional dyspepsia
Source: Front Pharmacol. 2022 Nov 17;13:1026660. doi: 10.3389/fphar.2022.1026660 (PMC9712737; doi:10.3389/fphar.2022.1026660)
Supplement: Supplementary file 1 [file Table1.docx]

**Table S1** Similarity of 10 batches of ZZKZ capsules.

| **Peak*** | ***Citrus Aurantium L.*** | ***Atractylodes Macrocephala Koidz.*** | ***Crataegus Pinnatifida Bunge*** | ***Bupleurum***  ***Chinense DC*.** |
| --- | --- | --- | --- | --- |
| 1 | + | - | - | - |
| 4 | - | - | + | - |
| 5 | - | - | - | + |
| 6 | + | - | + | + |
| 7 | + | - | - | + |
| 8 | + | - | - | - |
| 9 | + | - | - | - |
| 11 | + | - | - | - |
| 13 | + | - | - | - |
| 15 | + | - | - | - |
| 16 | + | - | - | - |
| 17 | + | - | - | - |
| 18 | + | - | - | - |
| 19 | + | + | - | - |
| 20 | + | + | - | - |

* The characteristic peaks in ZZKZ capsules that can be attributed in the four various herbs.
